# Recovery experiences and perceptions of quality of life after percutaneous coronary intervention for ST-elevation myocardial infarction: An interpretive descriptive qualitative study

**DOI:** 10.1371/journal.pone.0360291

**Published:** 2026-10-26

**Authors:** Fasaha F. Haji, Salome E. Buluba, Menti Lastone Ndile

**Affiliations:** 1 Department of Blood Donor Management, Ministry of Health Zanzibar, Zanzibar, Tanzania; 2 Department of Clinical Nursing, Muhimbili University of Health and Allied Sciences, Dar es Salaam, Tanzania; University of Diyala College of Medicine, IRAQ

## Abstract

**Background:**

Cardiovascular diseases remain the leading cause of mortality globally, with a disproportionate burden in low- and middle-income countries. Primary percutaneous coronary intervention is the standard treatment for ST-elevation myocardial infarction, a life-threatening acute coronary syndrome requiring urgent revascularization. Despite increasing access to this intervention in sub-Saharan Africa, evidence on patients’ lived experiences of recovery remains limited. This study explored patients’ experiences of recovery and quality of life following primary percutaneous coronary intervention for ST-elevation myocardial infarction in Tanzania.

**Methods:**

An interpretive descriptive qualitative design was employed. Fourteen patients who had undergone primary percutaneous coronary intervention for ST-elevation myocardial infarction were purposively sampled at a national referral cardiac centre and interviewed within three months after PCI. Data were collected through in-depth interviews, transcribed verbatim, and analyzed using reflexive thematic analysis.

**Results:**

Participants perceived quality of life after PCI as an evolving experience closely linked to their recovery, with improvements in one aspect often accompanied by ongoing challenges in others. Six interrelated themes emerged: physical symptoms, functional capacity and daily activities, emotional responses, coping and illness perceptions, social support and relationships, and health system interactions and communication. Although participants generally reported symptom improvement and gradual functional recovery, persistent fatigue, activity limitations, emotional uncertainty, and unmet informational needs continued to influence their quality of life. Family support and adaptive coping helped participants adjust to life after PCI, while healthcare communication shaped their confidence in managing recovery.

**Conclusion:**

Recovery after primary percutaneous coronary intervention extends beyond survival to include physical, emotional, and social dimensions. Holistic, patient-centred post-intervention care is needed to support long-term recovery in resource-limited settings.

## Introduction

Cardiovascular diseases (CVDs) remain the leading cause of death globally. In 2021, an estimated 20 million people died from CVDs, accounting for approximately 32% of all global deaths [1]. Over three-quarters of CVD-related mortality occurs in low and middle-income countries (LMICs) [2], reflecting significant inequities in prevention, diagnosis, and access to advanced cardiac care.

The burden of CVDs is particularly severe in sub-Saharan Africa (SSA), where CVDs account for approximately 13% of all deaths [3]. In Tanzania, CVDs are an emerging public health concern, also accounting for approximately 13% of all deaths and contributing substantially to morbidity, driven in part by rising rates of hypertension, diabetes, and other cardiovascular risk factors. According to the World Heart Federation, Tanzania recorded 56,908 CVD-related deaths in 2021, with an age-standardized mortality rate of 268 per 100,000 population, underscoring the magnitude of the national burden [4].

Coronary artery disease (CAD), a major subset of CVDs, results from the progressive accumulation of cholesterol and atherosclerotic plaque within the coronary arteries, leading to obstructive or non-obstructive lesions that impair myocardial perfusion [5]. Globally, CAD is the leading cause of cardiovascular mortality and contributes to nearly half of all CVD-related deaths and disability-adjusted life years [6]. Although precise prevalence estimates in SSA remain limited, available data indicate that CAD is among the three leading causes of adult mortality in many SSA countries [7].

Management of CAD includes coronary revascularization, most commonly through percutaneous coronary intervention (PCI). PCI is one of the most frequently performed cardiovascular procedures worldwide, with over two million interventions conducted annually [8]. ST-elevation myocardial infarction (STEMI) represents an acute manifestation of CAD, characterized by sudden coronary artery occlusion leading to myocardial necrosis and requiring urgent reperfusion therapy. Primary PCI is the preferred treatment strategy, as it rapidly restores coronary blood flow, limits infarct size, and improves survival when performed promptly [9].

In high-income countries, PCI services are supported by structured outpatient follow-up, comprehensive patient education, cardiac rehabilitation and psychosocial support. These services provide patients with guidance on physical activity, symptom recognition, medication adherence, risk-factor management, and psychological adjustment after PCI. In contrast, in Tanzania and many other SSA countries, access to PCI services remains limited, and post-procedural care often focuses more on acute clinical management and routine follow-up than on comprehensive rehabilitation and psychosocial recovery [10,11]. Structured cardiac rehabilitation programmes, individualized education, and systematic psychosocial support remain limited or inconsistently available. Therefore, patients may receive less guidance on management of the condition. These contextual differences are important because recovery after PCI extends beyond restoration of coronary blood flow and may influence overall quality of life.

Evidence from global studies indicates that PCI reduces mortality, prevents hospital readmissions, and improves quality of life (QoL) [12–17]. The World Health Organization defines QoL as a multidimensional construct encompassing individuals’ perceptions of their physical health, psychological state, level of independence, social relationships, and interactions with their environment [18]. However, most existing studies focus on clinical and health-related QoL outcomes, offering limited insight into how patients perceive and experience recovery within their sociocultural and healthcare contexts.

Exploring QoL through patients’ lived experiences is therefore essential for informing patient-centred cardiac care, optimising post-PCI follow-up, and guiding the development of psychosocial and rehabilitative interventions tailored to local needs. In response to this gap, this study aimed to explore patients’ lived experiences of recovery and QoL following primary PCI for STEMI at the Jakaya Kikwete Cardiac Institute (JKCI) in Dar es Salaam, Tanzania.

## Materials and methods

### Study design

This study employed an interpretive descriptive qualitative design, as described by Sally Thorne, to generate clinically meaningful insights grounded in patients’ experiences [19]. This approach is well-suited to exploring the complex and contextual nature of recovery and QoL following primary PCI for STEMI.

Interpretive description moves beyond surface description to identify patterns and meanings within subjective experiences through inductive and interpretive analysis. Unlike phenomenological approaches that seek to understand the essence of a shared lived experience, this study aimed to identify clinically relevant patterns across participants’ accounts that could inform post-PCI care.

Data collection and analysis were conducted iteratively, allowing emerging insights to inform ongoing inquiry. The study was reported in accordance with the Consolidated Criteria for Reporting Qualitative Research (COREQ) (Supporting Information, S1 Checklist).

### Study area

The study was conducted at JKCI in Dar es Salaam, Tanzania. The hospital functions as the nation's primary specialized cardiac centre, combining the roles of a university teaching hospital, a research institution, and a tertiary referral facility for Tanzania and neighbouring countries. The institute has a clinical capacity of approximately 150 inpatient beds and manages more than 1,800 outpatient visits weekly, with about 100 inpatient admissions. In terms of interventional cardiology services, its catheterization laboratory performs an average of 40–45 PCI procedures per month.

After discharge following PCI, patients are scheduled for their first follow-up visit within two weeks, where they are reviewed by cardiologists and nurses to assess their progress and receive further health education. Subsequent follow-up visits are scheduled according to the patient’s clinical progress and the outcome of the procedure. Currently, the hospital does not provide home-based follow-up care.

### Study population and eligibility

The study included adults (≥18 years) who had undergone primary PCI for STEMI within 3 months before the interview, ensuring that participants’ experiences reflected the early post-PCI recovery period.

This criterion ensured inclusion of participants who had experienced an acute, life-threatening coronary event requiring emergency revascularization, allowing for in-depth exploration of recovery following myocardial infarction. The three-month interval was selected to allow participants to move beyond the immediate post-procedural and acute recovery phase and to reflect meaningfully on their recovery and QoL.

Admission records were reviewed to screen and identify eligible patients with a documented diagnosis of STEMI. To minimize confounding influences on illness narratives, participants with major comorbid conditions or clinical events likely to dominate recovery experiences were excluded. These included active malignancy, end-stage renal disease requiring dialysis, major stroke with significant functional dependency, heart failure, recurrent MI, repeat PCI, or post-PCI complications, and severe chronic obstructive pulmonary disease. Patients who had undergone alternative revascularization procedures, such as coronary artery bypass grafting, were also excluded to ensure procedural consistency. Stable chronic conditions, including hypertension and diabetes mellitus, were permitted because they are common among patients with STEMI and, in the absence of major functional impairment, were not considered likely to dominate participants’ illness and recovery narratives.

### Sample size and sampling procedure

To enable an in-depth exploration of a shared experience, a purposive sampling strategy was employed, with homogeneity defined primarily by the clinical characteristics of the participants [20]. Specifically, all participants had experienced STEMI and undergone primary PCI within three months before the interview. This shared clinical experience provided a relatively homogeneous group in relation to the cardiac event and treatment received. Within this clinically defined group, variation in age, sex, recovery time following PCI, and social and educational background was intentionally sought to capture a range of perspectives on recovery and perceived QoL.

Participants were recruited during outpatient follow-up visits at the JKCI. Recruitment and data analysis were conducted concurrently to allow ongoing assessment of data adequacy and emerging patterns. All participants were interviewed within one month of the data collection period, ensuring temporal consistency in recruitment and that participants’ experiences remained within a comparable early post-PCI recovery period. During recruitment, four eligible participants declined participation because of lack of time due to other commitments. The final sample comprised participants with diverse demographic and social characteristics while maintaining the shared clinical experience of STEMI treated with primary PCI.

Data adequacy was assessed iteratively alongside data collection and analysis [21]. Following each interview, the researchers reviewed the data to identify emerging codes, concepts, and patterns and considered these in relation to the developing analysis from previous interviews. By the 12th interview, the dataset was providing sufficient depth and breadth to address the study objectives, and subsequent interviews largely reinforced the developing interpretations rather than substantially extending them. Interviews 13 and 14 were conducted to further assess the adequacy and richness of the dataset. These interviews provided additional perspectives that supported and elaborated the existing patterns without substantially altering the developing thematic interpretation. The core interview guide remained unchanged throughout data collection; however, probing questions were used flexibly to explore issues and experiences arising within individual interviews. Recruitment ceased after 14 participants when the researchers judged that the dataset contained sufficient depth, breadth, and richness to support the interpretive analysis.

### Data collection tool

The Data were collected through in-depth interviews using a semi-structured interview guide [22]. The interview guide (Supporting Information, S2 Text) was developed through a rigorous, multi-stage process. Initially, a comprehensive review of the literature informed the content of the guide followed by consultation with a subject matter expert to enhance the relevance, clarity, and depth of the questions. The WHO QoL framework was considered as a conceptual background for understanding QoL but was not used as an a priori framework for structuring the interview questions. The final guide comprised two sections: (1) participant background information, including key demographic and clinical characteristics, and (2) open-ended questions with non-leading prompts and probes exploring participants’ experiences of recovery and perceptions of QoL after PCI. The open-ended approach allowed participants to identify issues that were important to their experiences rather than restricting responses to predefined QoL domains.

The interview guide was initially developed in English and translated into Kiswahili by a bilingual researcher. The Kiswahili version was independently reviewed by two cardiovascular nurse specialists and refined based on their feedback. The revised interview guide was subsequently pre-tested with two patients who were not included in the main study. Feedback from the pre-testing was used to refine the wording and clarity of the interview questions. These procedures helped ensure that participants could understand the questions as intended and describe their experiences in their own words.

### Data collection process and management

Data collection was conducted from 14 April to 9 May 2025. Potential participants were initially approached during their scheduled clinic visits. The researcher explained the nature and purpose of the study. Those who expressed preliminary interest and met the eligibility criteria were invited to participate in an interview at a later time, scheduled after their clinical appointment to avoid interference with medical care.

Immediately before each interview, the researcher (FH) provided a detailed verbal explanation of the study aims, procedures, and key ethical considerations. These included assurances of confidentiality, the voluntary nature of participation, and the right to withdraw at any time without affecting their medical care. Written informed consent was then obtained, including permission for audio recording.

To ensure privacy, all interviews were conducted in a dedicated room separate from routine clinical activities. Interviews lasted between 30 and 50 minutes. Data were collected using audio recordings and supplemented with the researcher's field notes, which captured both verbal responses and relevant non-verbal cues.

Audio recordings were transcribed verbatim and anonymized by removing all personal identifiers. Transcripts and electronic field notes were stored on password-protected computers, while audio files and hard-copy materials were kept in a locked cabinet. Access to all study data was restricted to members of the research team.

### Data analysis

Data were analysed inductively in line with the principles of reflexive thematic analysis as outlined by Virginia Braun and Victoria Clarke [23]. This approach is guided by researcher reflexivity, flexible and iterative engagement with the data, and the understanding that themes are actively constructed through interpretation rather than simply discovered.

To preserve meaning, FH transcribed all Kiswahili audio recordings verbatim and conducted systematic, line-by-line coding in the original language to generate initial codes. The research team (FH, SB, MN) then collaboratively translated the codes into English. Codes and emerging patterns were discussed during iterative analysis meetings, where the team reflexively reviewed, refined, and organized potential themes. Themes and subthemes were then defined and named to reflect participants’ lived experiences of recovery and QoL.

In line with reflexive thematic analysis, coding and theme development were treated as iterative and interpretive rather than fixed or mechanical, with ongoing reflection on how the researcher's perspectives shaped analytic decisions. Analytical rigour was enhanced through continuous team discussions, constant comparison across interviews, and attention to coherence and consistency within and across themes. An independent qualitative research expert provided an external interpretive perspective through critical discussion of the developing analysis, enhancing the credibility and transparency of the analytic process. Direct participant quotations were selected to illustrate each theme and to ensure that findings remained grounded in participants’ accounts.

### Reflexivity

The research team reflected on how their professional backgrounds and positions may have influenced the research process. FH is a postgraduate student specializing in cardiovascular nursing while SB, and MN are nurse faculty with clinical experience in cardiovascular and critical care, respectively. The research team had no prior personal or professional relationship with the participants and were not involved in their clinical care. The clinical background of the research team facilitated rapport building and enhanced their understanding of participants’ medical narratives. However, this familiarity could also lead to assumptions and bias. To minimize this potential influence, FH, who conducted the interviews, practiced reflexivity and bracketing by intentionally setting aside clinical expectations and remaining open to participants’ experiences and perspectives. During analysis, the research team held regular discussions to critically examine and challenge emerging interpretations, ensuring that findings remained grounded in the data. The research team remained attentive to power dynamics and ethical responsibilities by emphasizing voluntary participation, ensuring confidentiality, and fostering respectful engagement throughout the study. Initial findings were shared with three participants who were purposively selected based on their availability and willingness to provide feedback. During an arranged meeting, participants were presented with a summary of the preliminary themes and their corresponding interpretations. They confirmed that the themes and interpretations generally represented their experience and therefore their feedback did not result in changes to the preliminary themes or overall analysis.

### Ethical considerations

Ethical approval was obtained from the Muhimbili University of Health and Allied Sciences (MUHAS) Ethical Research and Publications Committee (Ref: DA.282/298/01.C/2761). Permission to conduct the study at Jakaya Kikwete Cardiac Institute (JKCI) was granted by through the Head of Research, Training and Consultancy (Ref: AB.123/307/01L/62). Written informed consent, including consent for audio recording, was obtained from all participants. Confidentiality and anonymity were maintained, with participants identified only by study numbers.

## Results

### Participant characteristics

Fourteen participants were included in the study, with ages ranging from 55 to 75 years. Most participants were aged 60 years or older (11/14, 78.6%). The sample comprised equal numbers of men and women (7/14 each). The majority were married (11/14, 78.6%). Educational attainment varied across participants, with similar proportions having completed primary (5/14, 35.7%) or tertiary education (5/14, 35.7%). Stand-alone hypertension was the most commonly reported comorbid condition (8/14, 57.2%). Participant characteristics are presented in Table 1.

**Table 1:** Participant characteristics (N = 14).

Characteristic	n (%)
**Age (years)**	
<60	3 (21.4)
≥60	11 (78.6)
**Sex**	
Male	7 (50.0)
Female	7 (50.0)
**Marital status**	
Single	1 (7.1)
Married	11 (78.6)
Widowed	2 (14.3)
**Education level**	
Primary	5 (35.7)
Secondary	4 (28.6)
Tertiary	5 (35.7)
**Comorbid conditions**	
None	3 (21.4)
Hypertension only	8 (57.2)
Hypertension with diabetes	3 (21.4)

Six themes and their corresponding subthemes were generated, as presented in Table 2. Participants described recovery following PCI as a multidimensional experience that extended beyond the resolution of cardiac symptoms. Their perceptions of quality of life were shaped by changes in physical well-being, functional capacity, emotional state, coping and illness perceptions, social relationships, and interactions with the healthcare system. Six interrelated themes were identified: physical symptom experience; functional capacity and daily activity; emotional state and outlook; coping and illness perception; social support and relationships; and health system interaction and communication. Together, these themes demonstrate that participants’ perceptions of quality of life were closely intertwined with their recovery experiences. Improvements or challenges in physical and functional health, emotional well-being, social participation, coping, and interactions with the healthcare system shaped participants’ perceptions of their lives following PCI. Representative quotations are included in the Results section, with additional de-identified quotations supporting the identified themes provided in S3 Table.

**Table 2:** Themes, subthemes, and representative codes.

Theme	Subtheme	Representative Codes
Theme 1: Physical symptom experience	Relief and physical improvement	Relief from chest tightness, normalized heart rate, improved breathing, better sleep, regained weight, gradual physical recovery, improved mobility,
	Ongoing symptom burden and limitations	Fatigue, shortness of breath, dizziness, palpitations, headache, reduced exercise tolerance, increased need for rest.
Theme 2: Functional capacity and daily activity	Return to functional roles and independence	Gradual resumption of routine, independence in daily tasks, shift from employment to household roles, delegation of demanding tasks
	Activity restrictions and energy management	Reduced endurance, bed confinement during symptomatic periods, limited participation in physical activities
Theme 3: Emotional state and outlook	Positive emotional well-being and satisfaction	Feeling better, happiness, peace of mind, satisfaction with care
	Psychological distress and uncertainty	Anxiety, fear of recurrence, frustration, sadness, doubts about recovery, unmet expectations, disappointment with ongoing symptoms
Theme 4: Coping and illness perception	Adaptive coping and acceptance	Praying, reliance on faith/God, positive reframing, acceptance of condition, gratitude
	Hopeful future orientation	Positive outlook on life and treatment outcome, hope for extended life post-procedure, desire for regular checkups
Theme 5: Social support and relationships	Family and community support	Financial support from children, family involvement in care, peer consultation
	Social participation and role adaptation	Participation in social/religious/community events, retention of social/family roles
Theme 6: Health system interaction and communication	Positive engagement with healthcare	Good communication with providers, supportive hospital staff, confidence in medical advice given
	Communication gaps and unmet informational needs	Need for clearer explanations, difficulty reaching primary physician/ cardiologist, need to learn from experiences of other PCI patients, desire to ask questions after procedure

### Theme 1: Physical symptom experience

Participants frequently interpreted recovery in relation to survival from a life-threatening cardiac event. Improvements in symptoms were not only described as physical recovery but also carried symbolic meaning, reflecting relief at having “escaped death” and heightened awareness of bodily vulnerability.

#### Relief and physical improvement.

For many participants, percutaneous coronary intervention (PCI) was understood as restoring a sense of bodily normalcy through the alleviation of previously distressing symptoms such as chest discomfort, breathlessness, fatigue, and sleep disturbance. Symptom relief was widely taken as evidence of treatment success and contributed to a renewed sense of physical ease:

*“I feel much better after the procedure. Before, my heart was beating very fast, and I could not sleep at all.” (P04)“I feel better now. I no longer get tired as quickly, and my sleep has improved.” (P01)“The chest pain I had during the heart attack is gone, and I can breathe more freely now.” (P07)*

However, this improvement was not experienced as a complete return to pre-illness health. Rather, participants described a reduction in symptom burden that enabled greater participation in everyday activities while remaining conscious of ongoing bodily vulnerability. Thus, physical symptom relief was meaningful not only because participants felt better, but also because it expanded what they were able to do and supported a gradual return to familiar routines and roles.

#### Ongoing symptom burden and limitations.

At the same time, persistent fatigue and bodily weakness disrupted expectations of full recovery. Participants frequently described fatigue as unpredictable and disproportionate, suggesting a mismatch between perceived procedural success and lived bodily experience:

*“I feel tired… very tired… all the time.”* (P02)

*“My body gets tired easily. Even doing a small task makes me extremely exhausted.”* (P03)

*“Sometimes I feel weak even when I haven’t done much. I didn’t expect this after the procedure.”* (P09)

Persistent symptoms were experienced not only as bodily discomfort but also through their consequences for daily functioning. Fatigue, weakness, and reduced exercise tolerance restricted what participants could do and required them to modify routines, pace activities, or increase periods of rest. These experiences indicate that recovery following PCI involved ongoing negotiation with the body, in which improvement coexisted with lingering limitations.

Symptom persistence appeared to challenge expectations of cure, reframing recovery as management rather than resolution.

### Theme 2: Functional capacity and daily activity

Functional recovery was closely intertwined with participants’ physical symptom experiences. Improvements in symptoms enabled some participants to resume daily activities and reclaim independence, whereas persistent fatigue, weakness, and reduced exercise tolerance restricted activity and required adjustments to daily routines. Functional capacity therefore represented an important way through which participants experienced and interpreted their physical recovery.

#### Return to functional roles and independence.

For some participants, improvement in symptoms translated into a gradual reclaiming of routine roles and responsibilities. This return to activity was often interpreted as a marker of recovery and normalcy:

*“I’m now back to my normal life and doing my usual activities. There hasn’t been much change after the procedure.”* (P01)

*“After the procedure, I stayed for about three or four months without doing any heavy work. But now I’m slowly returning to my duties.”* (P05)

*“I can take care of myself again and move around without much assistance.”* (P10)

This progression highlights how recovery was experienced as a process of regaining autonomy, even if gradual and incomplete.

Regaining functional independence also carried emotional significance. Being able to resume familiar activities and responsibilities provided participants with tangible evidence that recovery was progressing and contributed to feelings of satisfaction, reassurance, and normalcy.

#### Activity restrictions and energy management.

In contrast, other participants described continued limitations that required them to actively regulate their energy and adjust their daily routines. Functional recovery, in this context, involved learning new limits and adapting behaviour accordingly:

*“I can’t do vigorous activities anymore. After a short time, I get tired and need to rest.”* (P08)

*“Cooking, washing dishes, going to the market, these things are still difficult for me.”* (P03)

*“I plan my day carefully. If I do too much, I suffer later.”* (P11)

These accounts suggest that recovery involved not only physical improvement but also recalibration of expectations and roles, with individuals negotiating a balance between activity and limitation. Importantly, these functional restrictions also had emotional consequences. The inability to perform previously routine activities, together with uncertainty about physical limits, could generate frustration, disappointment, and concern about whether recovery was progressing as expected. Functional limitation therefore represented both a physical constraint and an emotional experience of altered capability and independence

### Theme 3: Emotional state and outlook

Participants’ emotional responses were closely intertwined with their physical symptoms and functional capacity, reflecting how perceptions of bodily change and the ability to resume everyday activities shaped psychological well-being. Symptom relief and regained independence were generally associated with reassurance, satisfaction, and a sense of normalcy, whereas persistent symptoms and activity limitations contributed to anxiety, frustration, and uncertainty. Emotional responses also influenced how participants interpreted bodily sensations and approached their recovery.

#### Positive emotional well-being and reassurance.

Participants who experienced symptom relief often described a sense of reassurance and emotional stability. Improvement in physical symptoms appeared to restore confidence in their health and reduce anxiety:

*“I’m doing very well. I haven’t had any major problems, and I feel relaxed now.”* (P01)

*“I’m very happy with the treatment I received. It has really helped improve my condition.”* (P04)

*“Knowing that the procedure was successful gives me peace of mind.”* (P12)

These responses suggest that perceived treatment success contributed to emotional reassurance and a sense of regained control.

This reassurance was closely related to participants’ ability to perceive tangible improvements in their daily functioning; feeling physically capable of resuming ordinary activities appeared to provide additional evidence that recovery was progressing.

#### Psychological distress and uncertainty.

Conversely, when symptoms persisted, participants described ongoing distress, self-doubt, and uncertainty. Comparisons with others and unmet expectations of recovery intensified these concerns:

*“I am sad and not peaceful at all. I keep wondering if I will really get back to normal.”* (P02)

*“I keep asking myself, ‘Why haven’t I improved like others, even after treatment’”* (P03)

*“Sometimes I worry that something might still be wrong inside my heart.”* (P09)

These narratives reflect how incomplete recovery could generate uncertainty and perceived vulnerability. Persistent symptoms became particularly distressing when they interfered with everyday activities or contrasted with participants’ expectations of returning to normal life. Emotional distress was therefore not simply a response to symptoms themselves but emerged through the interaction between bodily sensations, functional limitations, and participants’ interpretations of what these changes meant for their recovery.

### Theme 4: Coping and illness perception

Participants actively constructed meaning around their experiences, drawing on personal, cultural, and social resources to manage ongoing challenges. Coping was particularly important when physical symptoms, functional limitations, or uncertainty generated emotional distress. Coping was not only behavioural but also interpretive, shaping how individuals understood their condition and recovery.

#### Adaptive coping and acceptance.

Faith, spirituality, acceptance and interpersonal support emerged as mechanisms through which participants made sense of their experiences and maintained emotional stability. Several participants drew on their faith to interpret their illness and recovery, particularly when their recovery did not fully match their expectations:

*“I thank God. I believe this is what God has planned for me.”* (P03)

For this participant, faith appeared to provide a framework for accepting the experience of illness and the uncertainty surrounding recovery. Similarly, another participant described accepting incomplete recovery following treatment:

*“I see some imperfections, but I accept them as part of the treatment consequences.”* (P07)

This acceptance appeared to help participants adjust their expectations and live with aspects of recovery that could not be immediately changed

Participants also described interpersonal and emotion-focused strategies for managing worries:

*“Talking to people and staying calm helps me manage my worries.”* (P10)

Thus, adaptive coping involved more than accepting the illness; participants actively used personal beliefs, acceptance, emotional regulation, and social interaction to manage concerns arising during recovery. However, these strategies were individual rather than necessarily shared by all participants, and the accounts do not establish that faith or spirituality directly influenced adherence to follow-up care.

#### Hopeful future orientation.

Despite ongoing challenges, many participants expressed hope for continued stability, while others adopted a more present-focused outlook shaped by uncertainty:

*“My wish is to remain healthy until my last day, without complications.”* (P04)

*“Now I just focus on attending my clinic appointments. Only God knows what will happen.”* (P03)

*“As long as I am stable, I am grateful.”* (P12)

These perspectives suggest that participants navigated recovery through a balance of hope, realism, and acceptance. Hope was not necessarily based on an expectation of complete restoration of pre-illness health; rather, it was often expressed as a desire to remain stable, maintain independence, and continue valued activities.

### Theme 5: Social support and relationships

Recovery was embedded within a broader social context, where family and community played a central role in shaping both practical and emotional experiences. Support systems functioned as enablers of recovery and buffers against vulnerability. Support systems functioned as enablers of recovery and buffers against vulnerability, particularly when physical or functional limitations affected participants’ ability to manage activities independently.

#### Family and community support.

Family members provided essential assistance with care, finances, and emotional reassurance, often influencing participants’ ability to access and sustain treatment:

*“My family supported me fully; they really care for me.”* (P02)

*“My daughter paid for the treatment. I wouldn’t have managed the cost on my own.”* (P06)

*“They always remind me to take my medicine and rest.”* (P11)

These accounts highlight the interdependence of recovery, where individual health experiences were closely linked to social support structures. Practical assistance could compensate for temporary or ongoing functional limitations, while emotional and informational support helped participants manage uncertainty and maintain confidence in their recovery.

#### Social participation and role adaptation.

Participants remained socially engaged, though often at a reduced level, reflecting efforts to maintain identity while accommodating physical limitations:

*“I still attend weddings and funerals, but I’m not as active as I used to be.”* (P05)

*“Before, I used to enjoy being in noisy or lively places, but now I have reduced that. These days, I prefer quieter places when I attend social gatherings”* (P06)

The ability to remain socially involved therefore depended partly on how participants negotiated their physical and functional limitations. Rather than representing complete withdrawal from previous roles, adaptation allowed participants to preserve social connections and a sense of belonging while modifying the level of participation to accommodate their changing capabilities.

### Theme 6: Health system interaction and communication

Participants’ experiences with the healthcare system influenced their understanding of recovery and their confidence in managing ongoing symptoms. While most participants described positive interactions with healthcare providers, some identified gaps in information and continuity of communication, particularly regarding medication use, persistent symptoms, activity restrictions, and expectations for longer-term recovery.

#### Positive engagement with healthcare providers.

Most participants expressed trust and satisfaction with healthcare providers, which contributed to reassurance and perceived quality of care:

*“The doctors have been very cooperative with me. I really appreciate the care they give me.”* (P01)

*“The nurses explained things well and treated me with respect.”* (P10)

Positive interactions appeared to strengthen participants’ confidence in the treatment received and supported their ability to make sense of recovery. Good communication therefore complemented physical and functional recovery by providing reassurance about symptoms, treatment outcomes, and ongoing care.

#### Communication gaps and unmet informational needs.

However, some participants described unresolved informational needs concerning persistent symptoms, medication use, and the expected course of recovery. For example, participants experiencing ongoing symptoms wanted clearer explanations about whether these symptoms were expected after PCI and when further assessment was necessary:

*“I really wish I could meet the doctor who did the procedure so he could explain this ongoing problem.”* (P08)

*“I ask why I still feel like this while others seem okay, but I don’t get clear answers.”* (P03)

Medication-related information was another area of uncertainty. Although participants understood the importance of continuing treatment, some remained unclear about the expected duration of medication use and what long-term ‘recovery’ meant:

*“I used to think healing meant getting better to the point of no longer using medication. But I was told that this is not the case, so I still wonder how long I will have to continue taking the medicine.”* (07)

These accounts indicate that the information needs extended beyond the procedure itself to include what to expect during recovery, which activities could safely be resumed, how to interpret persistent or new symptoms, possible medication side effects, how long medications should be continued, and when further clinical review was necessary. Thus, health system interaction influenced recovery not only through the clinical treatment provided but also through the availability, clarity, and continuity of information that enabled participants to interpret bodily changes and make decisions about activity, medication use, and follow-up care.

Overall, across the six themes, participants’ recovery experiences extended beyond the resolution of cardiac symptoms and reflected an ongoing process of adjustment. Physical symptom improvement enabled greater functional participation and independence, which in turn contributed to emotional reassurance and a sense of returning to normality. Conversely, persistent symptoms and functional restrictions could generate emotional distress and uncertainty, prompting participants to draw on coping strategies, social support, and healthcare interactions to interpret and manage these experiences. Recovery was therefore experienced as an interconnected process in which bodily changes, functional abilities, emotional responses, coping, social relationships, and healthcare interactions continually shaped one another.

## Discussion

This study explored the lived experiences of recovery and perceived QoL following primary PCI among patients with STEMI. In the context of the growing global and sub-Saharan African burden of cardiovascular disease [1–3] and the increasing access to interventional cardiology services in Tanzania [4,12]. The findings highlight an important distinction between procedural success and patients’ experience of recovery**.** Although primary PCI effectively addresses the acute coronary occlusion and provides substantial relief from ischaemic symptoms, participants described recovery as considerably more complex than restoration of coronary blood flow alone. Recovery emerged as a multidimensional, dynamic, and ongoing process involving physical symptoms, functional capacity, emotional responses, social relationships, coping, and interactions with the health system. Thus, from the patients’ perspective, successful PCI represented an important beginning of recovery rather than its endpoint.

Consistent with existing evidence, many participants described substantial relief of acute ischaemic symptoms, particularly chest pain and breathlessness, following primary PCI, underscoring its effectiveness as a life-saving intervention in the management of STEMI. These findings align with a broad body of literature demonstrating significant reductions in symptom burden and improvements in QoL after PCI across diverse settings [14–17]. However, an important finding in the present study was that relief of acute symptoms did not necessarily translate into a sense of complete recovery. Persistent fatigue emerged as a main concern, frequently described as disproportionate to activity levels and inconsistent with expectations of recovery. This aligns with prior studies identifying fatigue and reduced energy levels as common yet under-recognized post-PCI challenges [24,25]. The findings therefore suggest that conventional clinical indicators of PCI success, while essential, may not fully capture the broader dimensions of recovery that matter most to patients.

The prominence of fatigue offers an important lens for understanding the broader findings of this study. Participants did not define recovery solely in terms of whether chest pain had resolved or whether the procedure had been technically successful. Rather, recovery was closely linked to what they could do, how they felt, how they related to others, and whether they could resume their expected roles and responsibilities. In this sense, physical symptom experience formed a foundation for the subsequent dimensions of recovery identified in this study.

Fatigue may have important contextual implications especially in low-resource settings such as Tanzania, where livelihoods are often dependent on physical labour and daily productivity; unresolved fatigue may significantly disrupt income generation and household functioning. Evidence from other conditions suggests that fatigue is a key determinant of return to work and functional independence [26,27], and similar dynamics are likely to apply in post-PCI populations. In the absence of structured cardiac rehabilitation programmes, which remain limited in many LMICs, participants in this study appeared to navigate recovery largely independently, relying on self-developed strategies such as pacing and activity modification. This highlights a critical gap between successful acute intervention and the longer-term support required to achieve meaningful functional recovery and improved QoL.

Functional recovery varied considerably across participants, reflecting differences in individual capacity, social support, and adaptation strategies. While some participants regained independence and resumed routine activities, others experienced ongoing limitations requiring continuous adjustment. This variation further illustrates that recovery following PCI cannot be understood as a uniform or linear process. Rather, it reflects the interaction between physical capacity, individual interpretation of symptoms, available social support, and patients’ strategies for adapting to their post-PCI condition. This variability aligns with the self-regulation model of chronic illness, which conceptualizes patients as active agents who continuously interpret symptoms and adapt behaviours accordingly [28]. Recovery, therefore, is not a fixed endpoint but an evolving process shaped by ongoing interaction between the individual and their environment. These findings challenge standardized assumptions of uniform recovery patterns following PCI and underscore the need for more individualized, function-oriented rehabilitation approaches.

Emotional well-being was closely intertwined with physical recovery, with participants’ psychological states reflecting their interpretations of bodily change and treatment outcomes. Many participants expressed reassurance, gratitude, and renewed confidence following symptom relief, consistent with literature demonstrating the psychological benefits of successful cardiac interventions [29]. Gratitude, in particular, has been associated with improved mental health, resilience, and engagement in positive health behaviours [30,31]. However, this positive outlook frequently coexisted with anxiety, uncertainty, and self-doubt, particularly among those experiencing persistent symptoms or slower-than-expected recovery. These findings highlight the role of illness perception in shaping emotional outcomes, suggesting that recovery is not only physically experienced but also cognitively and socially constructed.

Coping strategies were strongly influenced by cultural and contextual factors. Faith, spirituality, and social connectedness emerged as central mechanisms through which participants made sense of their experiences and maintained emotional stability. This is consistent with a growing body of evidence highlighting the importance of spirituality and resilience in recovery from cardiovascular conditions [32–34]. For some participants in this study, these resources appeared to support emotional adjustment and adaptation to the challenges of recovery. However, these findings reflect the experiences of the participants in this study and should not be interpreted as representing the beliefs or coping practices of Tanzanian patients more broadly.

Family and social support were also described as important sources of emotional reassurance, practical assistance, and, in some cases, financial support during recovery. These accounts suggest that participants often drew on relationships with family members and others in their social networks when managing the challenges associated with recovery. Similar findings have been reported in previous qualitative studies of patients who underwent PCI, where social support has been associated with perceived safety, adherence, and recovery outcomes [35,36]. At the same time, reliance on family and social networks may create challenges for individuals who have limited or less reliable sources of support, potentially making aspects of recovery more difficult to navigate.

Despite generally positive perceptions of acute hospital care, participants identified important gaps in post-procedural support, particularly in relation to communication and continuity of care. Limited access to interventional cardiologists and insufficient explanations regarding persistent symptoms contributed to ongoing uncertainty and anxiety. These findings are consistent with previous research highlighting the importance of continuity of care and clear communication in improving patient outcomes after PCI [29,37]. The current findings suggest that health systems in resource-limited settings may be disproportionately focused on acute, procedure-based care, with insufficient attention to longer-term recovery support. The absence of structured follow-up and patient-centred education may leave patients to interpret ongoing symptoms independently, potentially undermining confidence and self-management.

Taken together, these findings indicate that recovery following PCI extends beyond biomedical restoration to encompass functional, emotional, and social dimensions that are insufficiently addressed within current care models. There is a need to reconceptualize post-PCI care as a continuum that includes structured follow-up, culturally responsive patient education, and support for functional and psychological recovery. In low-resource settings, this may involve strengthening multidisciplinary care, integrating community and family support systems, and developing context-appropriate rehabilitation strategies.

This study contributes to the existing literature by providing context-specific insights into recovery following PCI in Tanzania, where access to advanced cardiac interventions is expanding but post-procedural support systems remain limited. By foregrounding patient experiences, the findings highlight the need to align clinical definitions of success with patient-defined recovery, emphasizing a more holistic and patient-centred approach to cardiovascular care.

### Practical implications of the research findings

The findings highlight the need to extend PCI care beyond procedural success to address the multidimensional nature of recovery, including persistent physical symptoms, functional limitations, and psychological well-being. Routine, holistic post-PCI assessments should be incorporated into follow-up care to identify ongoing fatigue, reduced functional capacity, and emotional distress, which may otherwise remain under-recognised.

There is a clear need for context-appropriate rehabilitation strategies that support patients in managing energy levels, pacing activities, and gradually resuming daily roles, particularly in settings where formal cardiac rehabilitation services are limited. Strengthening post-discharge care through structured follow-up potentially led by nurses or community-based providers could improve continuity of care, enhance patient education, and support medication adherence and symptom management.

Improved communication between healthcare providers and patients is essential to address uncertainty regarding persistent symptoms and recovery expectations. Integrating psychosocial screening and incorporating family members into care planning may further enhance support systems, given the central role of family in patient recovery. A more coordinated, multidisciplinary, and patient-centred approach to post-PCI care is therefore critical to improving overall QoL in this population.

### Strengths and limitations

This study provides in-depth insights into patients’ lived experiences of recovery and QoL following PCI within a real-world, resource-limited setting, addressing an important gap in the predominantly quantitative literature. The use of an interpretive descriptive approach enabled a nuanced understanding of recovery from the patient perspective.

Several limitations should be considered. The study was conducted at a single national referral centre, which may limit the transferability of findings to other healthcare settings, particularly lower-level facilities. However, as a national referral centre, the site serves a geographically and socio-culturally diverse patient population, which allowed the study to capture perspectives from patients with varied backgrounds. The relatively small, purposively selected sample is consistent with qualitative methodology and prioritized depth over breadth, but may not reflect the full range of patient experiences.

Recruitment during scheduled outpatient follow-up may have introduced selection bias, as patients able to return to the national cardiac referral centre may have had better access to healthcare and fewer transportation, financial, or health-related barriers. Patients facing greater barriers to follow-up may therefore be underrepresented, potentially underestimating challenges related to continuity of care.

As with all interview-based research, responses may have been influenced by social desirability bias. To minimize this, participants were assured of confidentiality and encouraged to share their experiences openly. The cross-sectional design also captured experiences at a single point in time and could not capture changes in recovery and QoL over time; longitudinal studies are therefore warranted. Despite these limitations, the study provides rich, context-specific insights that contribute to a deeper understanding of post-PCI recovery and may inform more patient-centred care in similar settings.

## Conclusion

For patients with STEMI, primary PCI represents both a life-saving intervention and the beginning of a complex recovery journey. Recovery is shaped by persistent physical symptoms, emotional adjustment to a life-threatening event, and the availability of social support.

Improving long-term outcomes requires a shift toward a biopsychosocial model of care that integrates structured follow-up, patient education, and psychosocial support. Such an approach is essential to ensure that survival translates into meaningful improvements in QoL.

## Supporting information

S1 Checklist Consolidated Criteria for Reporting Qualitative Research (COREQ). (DOCX)

S2 Text Interview guide. (DOCX)

S3 Table Excerpts from interviews. (DOCX)
