# Supplementary material for: Recovery experiences and perceptions of quality of life after percutaneous coronary intervention for ST-elevation myocardial infarction: An interpretive descriptive qualitative study
Source: PLoS One. 2026 Oct 26;21(10):e0360291. doi: 10.1371/journal.pone.0360291 (PMC13704834; doi:10.1371/journal.pone.0360291)
Supplement: Supplementary file 1 [file pone.0360291.s001.docx]

**Consolidated Criteria for Reporting Qualitative Studies (COREQ)**

| **Item No** | | **Guide Questions/Description** | **Reported on Page #** |  |  |
| --- | --- | --- | --- | --- | --- |
| **Domain 1: Research team and reflexivity** | | | |  |  |
| **Personal Characteristics** | | | |  |  |
| 1. Interviewer/ facilitator | | Which author/s conducted the interview or focus group? | FH conducted the interviews (Pg 8) |  |  |
| 2. Credentials | | What were the researcher’s credentials? E.g., PhD, MD | FH and SB have MSc in Nursing, MN has PhD (Pg 9) |  |  |
| 3. Occupation | | What was their occupation at the time of the study? | FH was a student while SB and MN were university lecturers (Title page and Pg 9) |  |  |
| 4. Gender | | Was the researcher male or female? | Two females and one male (Title page (Title page and Pg 9) |  |  |
| 5. Experience and training | | What experience or training did the researcher have? | SB and MN have experience in Research Methodology during their careers as University teachers, while FH has experience during her studies and mentorship. |  |  |
| **Relationship with participants** | | | |  |  |
| 6. Relationship established | | Was a relationship established prior to study commencement? | No prior relationship existed between researchers and participants.(Pg 9) |  |  |
| 7. Participant knowledge of the interviewer | | What did the participants know about the researcher? e.g. personal goals, reasons for doing the research? | Participants were informed about the purpose of the research and their rights to participate. They were assured of privacy and confidentiality during and after the research (Pg 9&10) |  |  |
| 8. Interviewer characteristics | | What characteristics were reported about the interviewer/facilitator? e.g. Bias, assumptions, reasons and interests in the research topic | The researchers had a shared interest in the research (Pg 9) |  |  |
|  | **Domain 2: study design** | | |  |  |
|  | **Theoretical framework** | | |  |  |
|  | 9. Methodological orientation and Theory | What methodological orientation was stated to underpin the study? e.g. grounded theory, discourse analysis, ethnography, phenomenology, content analysis | The framework used was a Reflexive thematic analysis (Pg 8) |  |  |
|  | **Participant selection** | | |  |  |
|  | 10. Sampling | How were participants selected? e.g., purposive, convenience, consecutive, snowball | Participants were purposively selected (Pg 5) |  |  |
|  | 11. Method of approach | How were participants approached? e.g., face-to-face, telephone, mail, email | Participants were contacted physically and face-to-face interviews were conducted (Pg 6) |  |  |
|  | 12. Sample size | How many participants were in the study? | 14 participants took part in the study (Pg 7& 10) |  |  |
|  | 13. Non-participation Setting | How many people refused to participate or dropped out? Reasons? | Two contacted participants refused to participate due to a lack of time; All 14 participants who consented to participate did not drop out |  |  |
|  | 14. Setting of data collection | Where was the data collected? e.g., home, clinic, workplace | The data collection took place at the hospital during clinic visits (Pg 8) |  |  |
|  | 15. Presence of nonparticipants | Was anyone else present besides the participants and researchers? | No one was present besides the participants and researchers.(Pg 8) |  |  |
|  | 16. Description of sample | What are the important characteristics of the sample? e.g. demographic data, date | Age, sex, educational background, marital status, presence of comorbidity (Pg 5&6) |  |  |
|  | **Data collection** | | |  | No |
|  | 17. Interview guide | Were questions, prompts, and guides provided by the authors? Was it pilot tested? | The interview guide was rigorously reviewed and piloted. Follow-up questions/prompts were asked (Pg 7) |  |  |
|  | 18. Repeat interviews | Were repeat interviews carried out? If yes, how many? | No repeat interviews conducted |  |  |
|  | 19. Audio/visual recording | Did the research use audio or visual recording to collect the data? | The researcher used audio recording to collect data (Pg. 8) |  |  |
|  | 20. Field notes | Were field notes made during and/or after the interview or focus group? | Field notices were taken during the interview (Pg 8) |  |  |
|  | 21. Duration | What was the duration of the interviews or focus group? | The interviews lasted between 30 and 50 minutes (Pg 8) |  |  |
|  | 22. Data saturation | Was data saturation discussed? | Data saturation was discussed (Pg 6&7) |  |  |
|  | 23. Transcripts returned | Were transcripts returned to participants for comment and/or correction? | No transcripts returned |  |  |
|  | **Domain 3: analysis and findings** | | |  |  |
|  | **Data analysis** | | |  |  |
|  | 24. Number of data coders | How many data coders coded the data? | Three people participated in the coding process (Pg 8&9) |  |  |
|  | 25. Description of the coding tree | Did the authors provide a description of the coding tree? | Yes(Pg 11&12) |  |  |
|  | 26. Derivation of themes | Were themes identified in advance or derived from the data? | Derived from the data (Pg 8&9) |  |  |
|  | 27. Software | What software, if applicable, was used to manage the data? | Data were analyzed manually. |  |  |
|  | 28. Participant checking | Did participants provide feedback on the findings? | Initial findings were shared with some participants (Pg 9) |  |  |
|  | **Reporting** | | |  |  |
|  | 29. Quotations presented | Were participant quotations presented to illustrate the themes/findings? Was each quotation identified? e.g., participant number | Yes (Pg 13-19) |  |  |
|  | 30. Data and findings consistent | Was there consistency between the data presented and the findings? | Yes, the authors made sure to report the findings consistently reflecting on the collected data (Pg 12-25) |  |  |
|  | 31. Clarity of major themes | Were major themes clearly presented in the findings? | Yes (Pg 13-19) |  |  |
|  | 32. Clarity of minor themes | Is there a description of diverse cases or a discussion of minor themes? | Yes (Pg 13-19) |  |  |
