# Supplementary material for: Recovery experiences and perceptions of quality of life after percutaneous coronary intervention for ST-elevation myocardial infarction: An interpretive descriptive qualitative study
Source: PLoS One. 2026 Oct 26;21(10):e0360291. doi: 10.1371/journal.pone.0360291 (PMC13704834; doi:10.1371/journal.pone.0360291)
Supplement: Supplementary file 2 [file pone.0360291.s002.docx]

**Interview guide**

**Study title: Recovery and quality of life after percutaneous coronary intervention for ST-elevation myocardial infarction: Patients’ experiences from an interpretive description study**

**1. Opening and background**

- Can you tell me a little about yourself?
- Can you tell me about when you first became unwell?

**2. Experience of illness and treatment**

- Can you describe what happened when you first noticed something was wrong?
- Can you tell me about your experience of coming to the hospital and receiving treatment?

**3. Recovery experience**

- Can you tell me about your recovery since the procedure?
- How have things been for you over time?

**Probes:**

- What changes have you noticed?
- Has anything improved or become more difficult?
- Can you give an example?

**4. Daily life and activities**

- How has your condition affected your day-to-day life, if at all?
- What is a typical day like for you now?

**Probes:**

- Things you are able or unable to do
- Any changes in routine
- Ways you have adjusted

**5. Feelings and thoughts**

- How do you feel about your health now?

**6. Managing and coping**

- How have you been managing since your treatment?
- What helps you deal with your situation?

**Probes:**

- Things you do to manage day-to-day
- Sources of strength or support

**7. Support and relationships**

- In what ways, if any, have others been involved in supporting you?

**Probes:**

- Types of help received
- Any challenges

**8. Experiences with healthcare**

- Can you tell me about your experiences with healthcare since your treatment?
- How have follow-up visits or care been for you?

**Probes:**

- Communication with healthcare providers
- Information received
- Continuity of care

**9. Overall experience**

- Looking back, how would you describe your overall experience since your illness?

**Probes:**

- What has been most important
- What has been most challenging
- What has changed for you

**10. Closing**

- Is there anything else you would like to share about your experience?
- What would you want others to understand about going through this experience
