# Supplementary material for: Recovery experiences and perceptions of quality of life after percutaneous coronary intervention for ST-elevation myocardial infarction: An interpretive descriptive qualitative study
Source: PLoS One. 2026 Oct 26;21(10):e0360291. doi: 10.1371/journal.pone.0360291 (PMC13704834; doi:10.1371/journal.pone.0360291)
Supplement: Supplementary file 3 [file pone.0360291.s003.docx]

**THEMES, SUBTHEMES AND ANONYMIZED PARTICIPANT QUOTES**

| **Theme** | **Sub-theme** | **Quote** |
| --- | --- | --- |
| Theme 1: Physical Symptom Experience | Relief and physical improvement | “After the PCI, my chest feels open, and I can breathe easily.” |
|  |  | “Honestly, I can say that my quality of life has improved compared to before. Previously, I used to experience symptoms such as chest tightness, shortness of breath, and chest pain.” |
|  |  | “Things have improved because I can now do small tasks, and I also take evening walks every day. I walk about several kilometres, a total of a longer distance each day.” |
|  |  | “Aaah, I'm thankful. Up to today, I've been feeling much better. It's not like the condition I came here with.” |
|  |  | “I used to be breathless all the time, but now I breathe normally. I also had a dry cough without phlegm; it was persistent, but it has stopped.” |
|  | Ongoing symptom burden and limitations | “Sometimes when I work or walk a lot, my heart beats fast—like ‘pa pa pa pa!’ Sometimes I also become very itchy and have an allergic reaction. My body swells, especially my legs.” |
|  |  | “The difficulty I have now is actually from the medication. When I take it, my blood pressure drops, sometimes as low as a low level, and my body becomes weak. I also have no appetite, and sometimes I have trouble sleeping.” |
|  |  | “If something upsets me or I start thinking too much, I feel it in my heart. My heart starts racing, and I also become tired easily.” |
|  |  | “The drugs make me feel dizzy. When I take them, my head spins. I just wish my doctor could reduce the number of medications. I take many tablets in the evening alone, and with this body, that's too much.” |
| Theme 2: Functional Capacity and Daily Activity | Return to functional roles and independence | “After the procedure, I spent about some months without working. I had to rest and recover until I felt stable enough to resume my regular daily activities.” |
|  |  | “Yes, I managed to return to and continue with my work. However, there is still some fear in me, so my child also helps me with some of the work. It took about some time before I was able to return.” |
|  |  | “It took me some time. At first, I was afraid that working might interfere with my recovery.” |
|  | Activity restrictions and energy management | “I used to exercise regularly and was always jogging. But since becoming inactive because of this condition, that routine has decreased. When I exercise, I get tired. Even when I take my usual walks, I sometimes feel like sitting down.” |
|  |  | “When I bend over to scrub, I can't do it for long. I have to stop and rest because I feel exhausted.” |
|  |  | “I used to farm and manage my own work. Now I only do small tasks such as washing dishes or a few clothes. My ability to work is not the same. Once you've had such procedures, you can't be exactly as you were before.” |
| Theme 3: Emotional State and Outlook | Psychological distress and uncertainty | “Yes, you feel sad. You say to yourself, ‘Why is this happening now? I want to do this, but my body or my heart won't let me.’ When my blood pressure rises, I feel terrible.” |
|  |  | “After the surgery, I think I've become a bit more cautious and fearful. Before the surgery, I used to feel healthy. But once you're told you're sick, you start to feel pity for yourself.” |
|  |  | “I wonder how long I'll have to take these medications. regularly I have to come for a check-up, and you feel like you're wasting their time. Most of my stress comes from life issues, but obviously the treatment contributes too.” |
| Theme 4: Coping and Illness Perception | Adaptive coping and acceptance | “Staying idle because of illness is not a good thing. I just comfort myself.” |
|  |  | “My coping method is simply to take my medicine and rest; then I feel better again. We just surrender it all to God, our hearts, our blood pressure, and our whole bodies.” |
|  |  | “Mostly by talking, communicating with my doctor or my wife. Sometimes I go out for a walk, or I go to watch local activities to watch games. I also watch different TV programmes.” |
|  | Hopeful future orientation | “I think about my situation, but then I remind myself that others have even greater problems than mine. That helps me feel better.” |
|  |  | “I hope to live a better life, free from the difficulties I experienced before. When you can't even provide for yourself, you start feeling useless. So, I hope to live well, to be able to work and bring something home.” |
|  |  | “If my body gets stronger, I'll go back home. Even if I can't do much work, when someone sends me a little money, I manage, and life goes on.” |
| Theme 5: Social Support and Relationships | Family and community support | “After the procedure, my daughters, some of whom are married, talked to their husbands and came to stay with me. They looked after me. When it is time for clinic appointments, we go together. All of them help me, especially my children.” |
|  |  | “My children are my biggest support. My siblings, neighbours, and colleagues also help. They helped take me to the hospital, brought food, and checked on me during my recovery. I never lacked anything, not food and not care.” |
|  |  | “Since I got sick, people have become a bit closer to me. They check on me more often by calling to ask how I'm doing. I received support, especially when I first became ill. They even helped financially; I remember they contributed money for my transport to come here.” |
|  |  | “My children are the ones who care for me. They pay for my transport, food, and household needs. Who else would have brought me here? Even the ambulance from the referring area to here was paid for, it cost a lot. Without them, I wouldn't have made it.” |
|  | Social participation and role adaptation | “Relationships and communication? No. It's just the discomfort I feel. But no, I'm doing well with people. I have no issues.” |
|  |  | “I definitely attend community events, we help one another. When someone dies, we all contribute and attend the funeral. I participate fully in the community.” |
|  |  | “Since I got sick, people have become a bit closer to me. They check on me more often, calling to ask how I'm doing.” |
|  |  | “The only thing is that I don't like noise or getting annoyed. Noise makes me jumpy. Even if someone is shouting, I feel like my heart tightens, and I get startled.” |
| Theme 6: Health System Interaction and Communication | Positive engagement with healthcare providers | “Please continue offering this treatment to patients. Many people are happy when they hear about the cardiac centre. My peers say, ‘If you go to the cardiac centre, you're treated well.’” |
|  |  | “The treatment I received was excellent, and I was impressed by the doctors' competence.” |
|  | Communication gaps and unmet informational needs | “I really wish to meet the doctor who performed the PCI because we haven't met yet.” |
|  |  | “I'd just like to be monitored regularly to know whether I'm still okay. Going to the clinic and being given medication is routine, but I want to know how I'm really doing. That's the only thing that still concerns me.” |
|  |  | “I'm even thinking of asking the doctor to extend it to a longer interval because the transport cost is a challenge. I asked to be transferred [to a regional hospital], but they refused. I have to pay for my own transport and for the person who escorts me.” |
