# Supplementary material for: Recovery experiences and perceptions of quality of life after percutaneous coronary intervention for ST-elevation myocardial infarction: An interpretive descriptive qualitative study
Source: PLoS One. 2026 Oct 26;21(10):e0360291. doi: 10.1371/journal.pone.0360291 (PMC13704834; doi:10.1371/journal.pone.0360291)
Supplement: Supplementary file 4 [file pone.0360291.s005.docx]

**RESPONSE TO REVIEWERS/EDITORIAL COMMENTS**

1. **Editorial comments**

**Comment**: Please ensure that your manuscript meets PLOS ONE's style requirements, including those for file naming.

**Response:** Thank you for this comment. We have reviewed the manuscript and revised the files to ensure that they comply with PLOS ONE's style and file-naming requirements.

**Comment:** We note that this data set consists of interview transcripts. Can you please confirm that all participants gave consent for interview transcript to be published? If they DID provide consent for these transcripts to be published, please also confirm that the transcripts do not contain any potentially identifying information.

**Response:** Thank you for the comment. We clarify that the dataset consists only of de-identified information, including excerpts from the interview transcripts. All participants provided consent for their interview data to be used and published in de-identified form.

2. **Reviewer 1**

**Comment:** The study aim combines “recovery” and “quality of life,” but the findings do not clearly explain how each theme represents QoL. Please explicitly connect the six themes to QoL domains or clarify that the study focuses mainly on recovery experiences.

**Response**: Thank you for this important comment. We have clarified that the six themes collectively illustrate participants’ recovery experiences and perceptions of quality of life across physical, functional, psychological, social, and healthcare-related dimensions following PCI. The themes are interrelated rather than representing isolated aspects of the participants’ experiences. To make this relationship explicit, we added an introductory paragraph to the Results section (Pg 11, lines 261-274) explaining how the six themes relate to and collectively reflect participants’ perceptions of quality of life during recovery. We also revised the title to more accurately capture the study’s focus on both recovery experiences and perceptions of quality of life (See Title page).

**Comment:** The sampling process needs more detail. Please report how many eligible patients were approached, how many declined, and how purposive sampling ensured variation in age, sex, recovery time, and clinical background.
**Response:** Thank you for this important comment. We have clarified in the revised manuscript that the sample size was not predetermined and that recruitment was guided by data saturation, with recruitment and data analysis conducted concurrently. We have also expanded the description of the purposive sampling and recruitment process to clarify how participants were selected and how variation in age, sex, recovery time following PCI, and clinical background was considered within the homogeneous sample. In addition, we have clarified participant non-participation and the point at which recruitment was stopped based on thematic redundancy and confirmation of saturation. (See revised sample size and sample procedure section Pg 6&10)

**Comment:** The timing of interviews after PCI is unclear. Since patients were included at least 3 months after PCI, please report the exact range or median time from PCI to interview because experiences may differ greatly between 3 months and 1 year.

**Response:** Thank you for this observation. We apologize for the ambiguity in the wording. The phrase “at least 3 months before the interview” was intended to indicate that participants had undergone PCI within 3 months before the interview, rather than at least 3 months before the interview. This is also described in the subsequent paragraph. We have corrected this wording in the revised manuscript. See Pg 6&10.

**Comment:**The exclusion criteria mention major comorbidities, but the sample still includes hypertension and diabetes. Please clarify which comorbidities were allowed and whether heart failure, recurrent MI, repeat PCI, or post-PCI complications were considered.

**Response**: Thank you for this important observation. We have clarified the distinction between common, stable comorbidities and major comorbid conditions that could substantially influence participants’ recovery experiences. Stable chronic conditions, including hypertension and diabetes mellitus, were permitted because they are common among patients with STEMI and, in the absence of major functional impairment, were not considered likely to dominate participants’ illness and recovery narratives. In contrast, patients with major comorbid conditions or clinical events that could substantially affect recovery were excluded. These included heart failure, recurrent myocardial infarction (MI), repeat percutaneous coronary intervention (PCI), and major post-PCI complications. None of these conditions was present among the included participants. (Pg 6 Line 128-138)

**Comment:** The claim of data saturation needs stronger support. Please explain how saturation was assessed at the 12th interview and whether the interview guide changed as new themes emerged.

**Response:** Thank you for this important comment. We have expanded the description of how data saturation was assessed and attained. Recruitment and data analysis were conducted concurrently, and following each interview, the researchers reviewed the data for new codes, concepts, and emerging patterns. By the 12th interview, no new substantive codes or themes were emerging. Two additional interviews were conducted to confirm thematic redundancy and the adequacy of the dataset, and these did not yield any new substantive insights. The core interview guide remained unchanged throughout data collection; however, probing questions were used flexibly to explore new issues and obtain greater depth on participants’ experiences as they emerged during the interviews. (See Pg7 Line 156-157)

**Comment:**The methods mix interpretive description, reflexive thematic analysis, saturation, member checking, and independent expert review. Please explain how these approaches fit together methodologically, because some are not always aligned with reflexive thematic analysis.

**Response:** Thank you for the comment. We consider the interpretive descriptive design and reflexive thematic analysis to be methodologically complementary, with interpretive description providing the overall methodological orientation and reflexive thematic analysis providing the analytic approach. We have also reframed our descriptions of data saturation, participant feedback, and external qualitative review to better align with the study's interpretive and reflexive orientation. Specifically, we now describe data adequacy in terms of the depth, breadth, and richness of the dataset rather than as an objective point of saturation. Participant feedback and external qualitative review are described as opportunities for reflection and critical engagement with the developing interpretations, rather than as procedures for validating objectively correct themes. (See Pg7 Line 156-157 and Pg 8 & 9 Line 117-224)

**Comment:**- Some themes overlap, especially physical symptoms, functional capacity, and emotional state. Please strengthen the analysis by showing how these themes interact rather than presenting them as separate descriptive categories.

**Response:** Thank you for this important comment. We have strengthened the analysis by adding interpretive linking text throughout the findings to explicitly demonstrate the relationships between the themes. We have also added a synthesis paragraph highlighting the interconnections among the six themes, particularly how physical symptoms, functional capacity, and emotional well-being influence and shape one another during recovery. (See revised Result section)

**Comment:**Several subthemes are supported by very few quotations. Please add more participant quotations, especially for social participation, communication gaps, and coping, to better support the interpretation.

**Response**: Thank you for the comment. We have strengthened the findings by adding additional participant quotations to the subthemes, particularly social participation and communication gaps, to better support interpretation. (See Results section, Pg 18 & 19)

**Comment**: The health system communication theme is important but remains broad. Please specify what information patients lacked, such as activity limits, medication side effects, warning symptoms, follow-up plans, or when to contact a cardiologist.

**Response:** Thank you for this important comment. We have expanded the section to specify the areas in which participants experienced information gaps, including activity restrictions and safe resumption of activities, medication duration and potential side effects, interpretation of persistent or new symptoms, and continuity of follow-up and access to healthcare providers. (See Results section, Line 439-468)

**Comment:** The Data Availability statement says all relevant data are within the manuscript and supporting files. For qualitative interview data, please clarify whether de-identified transcripts are available or explain the ethical restrictions that prevent sharing full transcripts.

**Response:** Thank you for the comment. The available data include de-identified participant background information and selected de-identified excerpts from the interview transcripts that support the findings presented in the manuscript. All participants provided consent for their data to be shared in a de-identified format. Full interview transcripts are not publicly shared to protect participant confidentiality and privacy. (See Table 1 and Supporting Information, S3Table)

3. **Reviewer 2**

**Comment**: The Abstract is overly concise. I recommend including the time since PCI in the Abstract, as this information is highly relevant to the interpretation of the findings. Corresponding data should also be provided in the main text. In addition, the Results section of the Abstract currently reads more like a list of six separate domains. The authors should consider highlighting a central finding that cuts across these themes.

**Response:** Thank you for the comment. We have improved the abstract by adding more information, specifically time since PCI. We have also highlighted the central finding that participants experienced quality of life after PCI as an evolving experience closely linked to their recovery and how the themes interconnect rather than being presented as separate domains. (See revised Abstract Pg 2)

**Comment:** The difference in post-PCI support between high-income countries and Tanzania/sub-Saharan Africa is one of the most important contextual elements of this study. I recommend expanding this aspect more specifically in the Introduction**.**

**Response:** Thank you for the comment. We have expanded the Introduction to highlight the differences in post-PCI care between high-income countries and Tanzania, as well as other sub-Saharan African countries, with particular emphasis on access to structured follow-up, cardiac rehabilitation, patient education, and psychosocial support. (See revised Introduction Pg 4, Line 83-94)

**Comment:**3.The authors define QoL as encompassing physical health, psychological state, independence, social relationships, and the environment. However, the six themes subsequently identified—physical, functional, emotional, coping, social, and health-system domains—closely resemble these dimensions. The authors therefore need to clarify whether the WHO QoL framework was used only as conceptual background in the Introduction or whether it also informed the development of the interview guide or the data analysis. If the authors describe the analysis as fully inductive, they should explicitly state that these domains were not predetermined or used as an a priori coding framework; otherwise, readers may reasonably question whether the themes were organized according to an existing QoL framework.

**Response:** Thank you for the observation. We have clarified the role of the WHO quality of life framework in the study. The framework was used only as conceptual background to establish a broad understanding of QoL in the Introduction and was not used to develop the interview guide or to organize the data during analysis. The interview guide consisted of open-ended questions exploring participants’ experiences of recovery and perceptions of their quality of life, with probes used to explore issues raised by participants. (See Supporting Information file S2: Interview guide)

**Comment:4.** I recognize the regional value of this study, which is one of the main reasons I support major revision rather than rejection. However, there is no need to overstate its “global novelty.” The main contribution of this study is not the observation that patients may experience fatigue, anxiety, or dependence on family support after PCI. Rather, its value lies in showing how these experiences are perceived and managed within the specific context of social support, continuity of care, and rehabilitation resources in Tanzania. I therefore suggest shifting the emphasis from a “novel phenomenon” to a “context-specific understanding.”

**Response:** Thank you for this valuable comment. We have revised the Results and Discussion sections to place greater emphasis on participants' perceptions and management of their post-PCI experiences in the Tanzanian context. (See the revised Results and Discussion sections)

**Comment:** In the Methods section, the rationale for selecting interpretive description remains somewhat conceptual. The authors should provide additional explanation that the purpose of the study was not to identify a single phenomenological essence, but rather to identify clinically meaningful patterns across cases that could inform post-PCI care. This would make the methodological choice more convincing.
**Response:** Thank you for the comment. We have revised the Study Design section to provide a clearer rationale for selecting interpretive description in relation to the purpose of this study. Specifically, we clarified that the study did not aim to identify a single essence of the lived experience like phenomenological approaches, but rather to identify clinically meaningful patterns across participants’ experiences that could generate practical insights for post-PCI care. (See Study design, Pg 4, Line 100-103)

**Comment:** What is the usual care pathway for patients with STEMI after discharge following PCI in Tanzania? For example, do patients routinely see a cardiologist, is nurse-led follow-up available, and is there any formal cardiac rehabilitation program? This information would help readers interpret the findings more directly than hospital bed capacity alone.

**Response:** Thank you for the comment. Additional information has been provided in the setting section to include the care pathway for patients after discharge following PCI. (See Study Area, Pg 5 Line 114-118)

**Comment:** The rationale for including patients who had undergone PCI at least 3 months before the interview is understandable, but the absence of an upper limit and the actual distribution of time since PCI is an important gap that should be addressed. The authors explain that the 3-month threshold was chosen to allow patients to move beyond the most acute phase of recovery, which is reasonable. However, it is currently unclear whether the 14 participants were interviewed 3 months, 6 months, or several years after PCI. Given that the study focuses on “recovery,” the authors should report the median and range of time since PCI, or at least provide meaningful categories. If the interval varies substantially across participants, this temporal heterogeneity should also be considered in the analysis and discussed in the Limitations.

**Response:** Thank you for the comment. We would like to clarify that the intended inclusion criterion was that patients were interviewed within three months after PCI, rather than at least three months after PCI. The previous wording was unclear and may have suggested an open-ended period extending several months or years after PCI. We have made a correction in methods section. (See Study population Pg 6, Line 137-139)

**Comment**: “Homogeneous purposive sampling” is a reasonable strategy, but the authors should explain more clearly which characteristics were intended to be homogeneous and which dimensions were allowed to vary. All participants had experienced STEMI and undergone primary PCI, which clearly defines a shared clinical experience. However, the final sample included equal numbers of men and women and considerable variation in educational background. The authors should clarify whether variation in sex, age, or social background was intentionally sought during recruitment or whether this distribution arose naturally. This would make the sampling logic easier to understand**.**

**Response:** Thank you for the observation. We have revised the description of the sampling strategy to clarify that homogeneity referred primarily to the participants’ shared clinical experience of STEMI treated with primary PCI within three months before the interview. Within this clinically homogeneous group, variation in age, sex, recovery time following PCI, and social and educational background was intentionally sought to capture diverse perspectives on recovery and perceived QoL. (See Study population Pg 6, Line 161-166)

**Comment:** Recruitment during scheduled outpatient follow-up introduces a potential selection issue that should be explicitly acknowledged. Patients who are able to return to a national cardiac referral center for scheduled follow-up may have better access to care than those who are lost to follow-up, face transportation or financial barriers, or have poorer physical health. As a result, the study may underestimate the true extent of continuity-of-care barriers. This does not diminish the value of the study, but it should be transparently acknowledged in the Limitations. In research from resource-limited settings, I believe an honest account of such contextual limitations is more informative than attempting to imply broad representativeness.

**Response:** Thank you for the comment. We have revised the Strengths and Limitations section to clearly acknowledge the potential selection bias associated with recruiting patients who attended scheduled outpatient follow-up. We have clarified that patients unable to attend follow-up have not been represented, potentially resulting in an underestimation of the true extent of continuity-of-care barriers.. (See Pg 25, Line 623-629)

**Comment:**The description of “saturation” at the 12th interview, followed by two additional interviews for confirmation, should be made more consistent with the authors’ use of reflexive thematic analysis. The manuscript states that no new codes or themes emerged and that thematic redundancy was therefore reached at interview 12, after which two additional interviews were conducted. This is a common formulation in conventional qualitative research, but it creates some tension with the later statement that “themes are actively constructed through interpretation rather than simply discovered.” Given the context of this study, I would mainly suggest revising the terminology toward data adequacy or information richness. For example, the authors could state that by approximately the 12th interview, the dataset had sufficient depth and diversity to address the research question, and that two additional interviews were conducted to assess the adequacy of the developing interpretation. This would preserve the existing study design while aligning the language more closely with the chosen analytic approach.

**Response:** Thank you for the comment. We have revised the description of data saturation to state that two additional interviews, the 13^th^ and 14^th^, were conducted after the 12th interview to assess data adequacy. Sufficient depth and diversity of information had been achieved by the 12th interview.. (See Pg 6 &7, Line 156-167)

**Comment:** In describing the development and refinement of the interview guide, I suggest avoiding terminology such as “validity,” which is more commonly associated with instrument or scale development. It would be more appropriate to explain directly how these procedures improved the clarity, cultural appropriateness, and non-leading nature of the interview questions.

**Response:** Thank you for the comment. We have revised this section to use more appropriate, commonly used terminology in qualitative research when describing the development, translation, and refinement of the interview guide. We have also clarified how the review and pre-testing processes improved the final version of the interview guide.(See Pg 7, Line 180-186)

**Comment:** The authors should clarify whether the two patients who provided feedback on the interview guide were later included in the formal study sample. If they were involved only in testing the wording and comprehensibility of the questions but subsequently participated in the interviews, the authors should consider whether their prior exposure to the interview questions may have influenced their responses.

**Response:** Thank you for the comment. We clarified that two patients involved in pretesting of the interview guide did not participate in the main study; rather, they were involved in providing feedback on the interview guide before the commencement of the main study. (See Pg 7, Line 182-189)

**Comment:** More information should be provided about the interviewer, FH, including professional background, whether FH was a clinical staff member at JKCI, whether FH had been involved in the care of any participants, and whether participants knew FH before the interviews. This is particularly relevant because many participants later expressed positive views of doctors and nurses. The interviewer’s relationship with the healthcare system may have influenced participants’ willingness to express criticism.

**Response:** Thank you for the comment. We have provided more background of FH who was involved in the interview process and measures used to minimize potential social desirability bias or power dynamics. (See Reflexivity section Pg 9)

**Comment:** The statement that “Initial findings were shared with selected participants” requires further detail. The authors should specify how many participants were involved, how they were selected, whether they were shown preliminary themes or a summary of the findings, what type of feedback they provided, and whether this feedback led to any changes in the analysis.

**Response:** Thank you for the comment. We have clarified that three participants were involved in providing feedback on the initial analysis. They confirmed that the interpretations represented their experiences and therefore no changes were made to the subsequent analysis and interpretation. (See Reflexivity section Pg 9, Line 239-243)

**Comment:** Theme 1 is currently the strongest part of the manuscript, and I suggest elevating its interpretation into the central thread of the paper. The subsequent Results and Discussion could be more explicitly connected to this idea: PCI addressed the acute coronary occlusion, but patients’ understanding of “recovery” was considerably more complex than procedural success alone.

**Response:** Thank you for the comment. We have expanded the interpretation of Theme 1 in both the Results and Discussion sections and strengthened its role as a central interpretive theme of the study. (See Results section, Pg. 12- 13 and Discussion section, Pg 21-22, Line 525-537)

**Comment**: The role of faith and spirituality in Theme 4 is particularly valuable in the Tanzanian context, but the current analysis is somewhat brief. Participants used references to God, acceptance, and gratitude to make sense of their illness and maintain emotional stability. The authors could explore these functions in greater depth—for example, whether faith and spirituality helped patients accept incomplete recovery, reduce uncertainty, or maintain engagement with follow-up care. At the same time, the discussion should avoid reducing African patients to overly “cultural” or “religious” explanations and should remain grounded in the participants’ own accounts.

**Response:** Thank you for the comment. We have expanded the analysis of Theme 4 to provide greater depth on the roles of faith, spirituality, acceptance, and interpersonal coping in helping participants make sense of their illness and manage the challenges of recovery. We have also clarified that, although these strategies supported participants in coping with their recovery experiences, no direct influence on follow-up care was established from the participants’ accounts. (See Theme 4 in the Results section Pg 16 & 17)

**Comment:** There are several unusual typographical and editorial errors in the manuscript, such as “improvements in q after PCI.” The manuscript should be carefully proofread. There is also an inconsistency between “Fasaha F. Haji” and “Fasaha F. Hajji.” It is particularly important to avoid errors in the authors’ own names.

**Response:** Thank you for the observation. We have carefully proofread the manuscript and corrected the identified typographical errors

**Comment:** The discussion of spirituality and the “collectivist social context” is reasonable, but the authors should avoid extrapolating from family support reported by a small number of participants to broader conclusions about the culture as a whole.

**Response:** Thank you for the comment. We have revised this section to emphasize that the findings reflect the individual experiences of participants rather than generalizing from the experiences of a small number of participants to the broader cultural context. We have also clarified that family and social support were important resources for some participants without attributing these experiences to a generalized collectivist social context. (See Pg 22. Line 548-555)
